# Southern Doctors and Their Work

**Published:** 1890-10

**Authors:** 


### SOUTHERN DOCTORS AND THEIR WORK.

---

Now and then we learn, in private conversation, that Dr. So-and-So has done some especially skilful surgical operation, or has had a series of interesting or rare cases, or has accurately observed some symptom, or some action of a drug which would prove of interest to the profession at large. Still many of our Southern doctors plead want of time as their excuse for not writing up their experience. They forget that the doctor who writes and carefully reports his cases is the one who becomes known outside of his own locality, and is the one who may add one solid stone to the fabric of medicine, which shall be as a monument to his skill after he is dead. A well posted doctor recognizes what is new, and need not fear that any carefully prepared article will fall below the average. A great deal of "trash" is written, but our Southern doctor need have no fear that his will be a greater share than emanates from our Northern, German, French or English brethren.

A prominent young practitioner said the other day, that those fellows up North always let us know when they do anything, which reminds us of the lawyer who said of a district judge, that he made a great "to-do" about his decisions, like a hen running out cackling after laying an egg. Much of the doctors' work may appear as ordinary and routine as the hen's laying, but other parts are rare and unique enough to justify a little "cackling." So, doctors, wherever you live and however modest you be, you can help the Southern profession, help yourself, and show our bright and energetic friends of the North that something good *can* "come out of Nazareth."

